# Differential diagnosis of pleural mesothelioma using Logic Learning Machine

**DOI:** 10.1186/1471-2105-16-S9-S3

**Published:** 2015-06-01

**Authors:** Stefano Parodi, Rosa Filiberti, Paola Marroni, Roberta Libener, Giovanni Paolo Ivaldi, Michele Mussap, Enrico Ferrari, Chiara Manneschi, Erika Montani, Marco Muselli

**Affiliations:** 1 Institute of Electronics, Computer and Telecommunication Engineering, National Research Council of Italy, Via De Marini, 6, 16149 Genoa, Italy; 2 Epidemiology, Biostatistics and Clinical Trials, IRCCS AOU San Martino-IST, L.go R. Benzi, 10, 16132 Genoa, Italy; 3 Laboratory Medicine Service, IRCCS AOU San Martino-IST, L.go R. Benzi, 10, 16132 Genoa, Italy; 4 Pathology Unit, Azienda Ospedaliera Nazionale SS. Antonio e Biagio e Cesare Arrigo, Via Venezia 16, 15121 Alessandria, Italy; 5 Department of Pneumology, AO Villa Scassi, Corso Scassi, 1, 16149 Genoa, Italy; 6 IMPARA Srl, Piazza Borgo Pila 39, 16129 Genoa, Italy

## Abstract

**Background:**

Tumour markers are standard tools for the differential diagnosis of cancer. However, the occurrence of nonspecific symptoms and different malignancies involving the same cancer site may lead to a high proportion of misclassifications.

Classification accuracy can be improved by combining information from different markers using standard data mining techniques, like Decision Tree (DT), Artificial Neural Network (ANN), and *k*-Nearest Neighbour (KNN) classifier. Unfortunately, each method suffers from some unavoidable limitations. DT, in general, tends to show a low classification performance, whereas ANN and KNN produce a "black-box" classification that does not provide biological information useful for clinical purposes.

**Methods:**

Logic Learning Machine (LLM) is an innovative method of supervised data analysis capable of building classifiers described by a set of intelligible rules including simple conditions in their antecedent part. It is essentially an efficient implementation of the Switching Neural Network model and reaches excellent classification accuracy while keeping low the computational demand.

LLM was applied to data from a consecutive cohort of 169 patients admitted for diagnosis to two pulmonary departments in Northern Italy from 2009 to 2011. Patients included 52 malignant pleural mesotheliomas (MPM), 62 pleural metastases (MTX) from other tumours and 55 benign diseases (BD) associated with pleurisies. Concentration of three tumour markers (CEA, CYFRA 21-1 and SMRP) was measured in the pleural fluid of each patient and a cytological examination was also carried out.

The performance of LLM and that of three competing methods (DT, KNN and ANN) was assessed by leave-one-out cross-validation.

**Results:**

LLM outperformed all other considered methods. Global accuracy was 77.5% for LLM, 72.8% for DT, 54.4% for KNN, and 63.9% for ANN, respectively. In more details, LLM correctly classified 79% of MPM, 66% of MTX and 89% of BD. The corresponding figures for DT were: MPM = 83%, MTX = 55% and BD = 84%; for KNN: MPM = 58%, MTX = 45%, BD = 62%; for ANN: MPM = 71%, MTX = 47%, BD = 76%.

Finally, LLM provided classification rules in a very good agreement with *a priori *knowledge about the biological role of the considered tumour markers.

**Conclusions:**

LLM is a new flexible tool potentially useful for the differential diagnosis of pleural mesothelioma.

## Background

Differential diagnosis of cancer plays a crucial role in addressing medical therapies and surgical interventions. However, cancer diagnosis can become a very difficult task in the presence of nonspecific symptoms and different malignancies involving the same cancer site.

Malignant pleural mesothelioma (MPM) is a rare highly fatal tumour, whose incidence is rapidly increasing in developed countries due to the widespread past exposure to asbestos in environmental and occupational settings [1]. The correct diagnosis of MPM is often hampered by the presence of atypical clinical symptoms that may cause misdiagnosis with either other malignancies (especially adenocarcinomas) or benign inflammatory or infectious diseases (BD) causing pleurisies [2]. Cytological examination (CE) may allow to identify malignant cells, but sometimes a very high false negative proportion may be encountered due to the high prevalence of non-neoplastic cells [2]. Moreover, in most cases a positive result from CE only does not allow to distinguish MPM from other malignancies [3].

Many tumour markers (TM) have been demonstrated to be useful complementary tools for the diagnosis of MPM [4-6]. In particular, a recent investigation, based on pairwise comparisons by standard ROC analysis, analysed the concentrations of three tumour markers in pleural effusions, namely: the soluble mesothelin-related peptide (SMRP), CYFRA 21-1 and CEA, and their association with a differential diagnosis of MPM, pleural metastasis from other tumours (MTX) and BD [7]. SMRP showed the best performance in separating MPM from both MTX and BD, while high values of CYFRA 21-1 were associated to both MPM and MTX. Conversely, high concentrations of CEA were mainly observed in patients with MTX. Taken together, these results indicate that information from the three considered markers and from CE might be combined together in order to obtain a classifier to separate MPM from both MTX and BD.

Logic Learning Machine (LLM) is an innovative method of supervised data mining able to provide threshold-based rules for classification purposes [8,9]. The present investigation is aimed at illustrating the application of LLM for the differential diagnosis of MPM by identifying simple and intelligible rules based on CE and TM concentration. Preliminary results of the present study have been published as an extended abstract in the framework of the Bioinformatics Italian Society annual meeting 2013 [10].

## Methods

### Data set description

A consecutive cohort of 177 patients admitted for diagnosis to two pulmonary departments in Northern Italy from 2009 to 2011 was considered as eligible. Concentration of SMRP, CYFRA 21-1 and CEA tumour markers was measured in pleural effusion as described by Filiberti et al. [7].

All patients underwent CE, while 8 had at least one missing data for a considered TM, and were consequently excluded from the study, thus leaving 169 patients available for the analyses (namely: 52 MPM, 62 MTX and 55 BD). Study design was carried out according to the protocol "Research on pulmonary diseases" approved by the ethical committee of AO Villa Scassi Hospital of Genoa, Italy, on 15 December 2005.

An informed consent for analysis of pleural fluid was obtained from all patients.

Descriptive statistics of the three considered TM and results of CE are resumed in Table 1. SMRP concentration was higher among MPM than in the other two classes, whereas CYFRA 21-1 showed very low values among BD and higher values among the two malignancies, with the highest median concentration observed for MPM. CEA showed high values among MTX and similar low values among the other two classes. The corresponding interquartile ranges were largely overlapping, indicating that no considered TM can provide a perfect separation between MPM and the other two classes. Finally, CE provided a positive result in about one third of MPM and a half of MTX patients only, confirming the very low sensitivity of such technique [2]. Furthermore, a positive CE result was observed among BD, which corresponded to a very old patient who died after a short period of follow-up, as described in Filiberti et al. [7]. It remains unclear if it was due to the occurrence of some latent pleural malignancy or it actually represents a false positive result.

**Table 1:** Characteristics of 169 patients with pleural disease according to benign and malignant pleural effusion.

Diagnosis	SMRP (nmol/l) Median *(IQR)*	CYFRA 21-1 (ng/l) Median *(IQR)*	CEA (ng/l) Median *(IQR)*	Cytology (% of positivity)
MPM	24.2 *(8.8-55.0)*	226.6 *(117.6-732.2)*	0.9 *(0.5-1.5)*	32.7
MTX	4.6 *(2.6-10.0)*	120.9 *(41.7-446.7)*	8.5 *(1.4-72.8)*	50.0
BD	2.8 *(1.0-7.5)*	23.1 *(7.8-48.8)*	1.1 *(0.5-1.7)*	1.8

MPM = Malignant Pleural Mesothelioma; MTX = Metastasis; BD = Benign Disease (pleurisis); IQR = Interquartile Range.

### LLM classification rules

Information from tumour marker concentrations and CE was combined using a set of simple intelligible rules, automatically generated by the LLM algorithm, which is an efficient implementation of the Switching Neural Network model [8]. In more details, let ***x ***∈ℜ*^d ^*be a *d*-dimensional example in a classification problem to be assigned to one of *q *possible classes, labeled by the values of a categorical output *y*. Starting from a *training set S *including *n *pairs (***x***_i_,y_i_), *i *= 1,..., *n*, deriving from previous observation, LLM has the aim of generating a *classifier*, *i.e*. a model *g*(***x***) that provides the correct answer *y *= *g*(***x***) for most input patterns ***x***. Concerning the components *x_j _*two different situations can be devised: a) ordered variables: *x_j _*varies within an interval [*a*,*b*] of the real axis and an ordering relationship exists among its values; b) nominal (categorical) variables: *x_j _*can assume only the values contained in a finite set and there is no ordering relationship among them. LLM generates an intelligible model *g*(***x***) described by a set of *m *rules *r_k_*, *k *= 1,..., *m*, in the **if-then **form:

if<premise>then<consequence>

where <premise> is the logical product (AND) of *m_k _*conditions *c_kl_*, with *l *= 1,..., *m_k_*, on the components *x_j_*, whereas <consequence> gives a class assignment *y *= *ỹ *for the output. In general, a condition *c_kl _*in the premise involving an ordered variable *x_j _*has one of the following forms *x_j _*>*λ*, *x_j _*≤ *µ*, *λ *<*x_j _*≤ *µ*, being *λ *and *µ *two real values, whereas a nominal variable *x_j _*leads to membership conditions *x_j _*∈ {*α*, *δ*, *σ*}, being *α*, *δ*, *σ *admissible values for the *j*-th component of ***x***.

For instance, if *x*_1 _is an ordered variable in the domain [1,100] and *x*_2 _is a nominal component assuming values in the set {*A, B, C*}, a possible rule *r*_1 _is:

$$if\;x_{\text{1}}>\;40\;and\;x_{\text{2}}\epsilon \left\{A,B\right\}\;then\;y=0$$

where 0 denotes one of the *q *possible assignments (classes).

### The LLM algorithm for rule extraction

**Figure 1:**
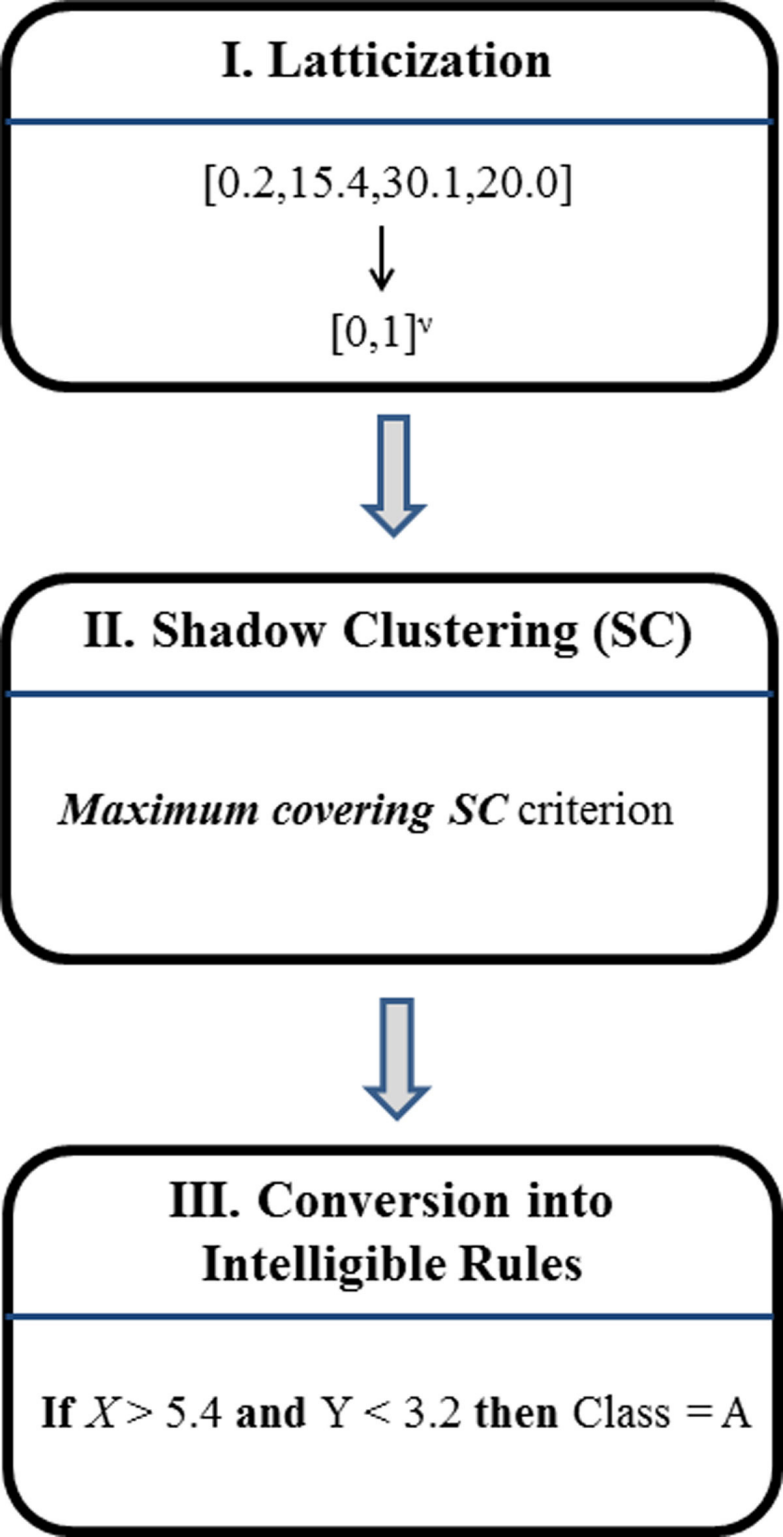
**The three steps of Logic Learning Machine**.

Intelligible classification rules described in the previous paragraph are generated by LLM following the three steps illustrated in Figure 1. During the first step (latticisation or binarisation), data are binarized according to the inverse only one code, which allows to preserve ordering and distance when used to transform both ordered and nominal attributes [8].

By means of binarisation, each example is therefore transformed into a string ***z *∈**{0,1}*^v ^*of binary values (bits). The length of these strings (i.e. the number of bits), denoted by *v*, depends on the number of inputs and on the number of values that each input assumes in the training set. After this step the training set has been translated into a binary input-output matrix which can be seen as a portion of the truth table of a monotone Boolean function.

The second step adopts a proper technique for digital synthesis capable of retrieving in a reasonable time a monotone Boolean function consistent with a partially described truth table. A method of this kind is the Shadow Clustering (SC) algorithm [9], which builds at each iteration a new logical product (implicant) to be added to the final AND-OR expression for the monotone Boolean function and adopts specific approaches to increase the classification ability of each produced implicant.

In particular, the Maximum covering Shadow Clustering criterion [9] attempts to increase the number of training patterns covered by each implicant while keeping low its complexity. Extensive trials has shown that the resulting procedure leads to excellent intelligible models in the analysis of several real-world classification problems [9].

Finally, in the third step from every generated implicant an intelligible rule, including a logical product (AND) of simple conditions, is automatically retrieved. The resulting set of rules forms the classification model for the problem at hand.

More details about SC implementation and estimates of the related computational burden under different scenarios are provided in dedicated papers [8,9].

### Quality measures of LLM and Class Prediction

According to the output value included in their consequence part, the *m *rules *r_k _*describing a given model *g*(***x***) can be subdivided into *q *groups *G*_1_, *G*_2_,..., *G_q_*. Considering the training set *S*, any rule *r *∈ *G_l _*is characterized by four quantities: the numbers True Positive (*TP*(*r*)) and False Positive (*FP*(*r*)) of examples (***x***_i_,y_i_) with *y_i _*= *y_l _*and *y_i _*≠ *y*_l_, respectively, that satisfy all the conditions in the premise of *r*, and the numbers False Negative (*FN*(*r*)) and True Negative (*TN*(*r*)) of examples (***x***_i_,y_i_) with *y_i _*= *y_l _*and *y_i _*≠ *y*_l_, respectively, that do not satisfy at least one of the conditions in the premise of *r*.

Starting from *TP(r)*, *FP(r)*, *TN(r)*, and *FN(r)*, other useful characteristic quantities, such as the *covering C*(*r*), the *error E*(*r*), and the *precision P*(*r*) can be derived:

(1) $$C\left(r\right)=\frac{TP\left(r\right)}{TP\left(r\right)+FN\left(r\right)}$$

(2) $$E\left(r\right)=\frac{FP\left(r\right)}{TN\left(r\right)+FP\left(r\right)}$$

(3) $$P\left(r\right)=\frac{TP\left(r\right)}{TP\left(r\right)+FP\left(r\right)}$$

*C*(*r*) and *P*(*r*) are also known as the Sensitivity and the Positive Predictive Value in Clinical Epidemiology setting, while E(r) corresponds to 1 - Specificity.

*C*(*r*) and *P*(*r*) are usually adopted as measures of relevance for a rule *r*. As a matter of fact, the greater is the covering and the precision, the higher is the generality and the correctness of the corresponding rule.

On the other hand, to obtain a measure of relevance *R*(*c*) for a condition *c *included in the premise part of a rule *r*, one can consider the rule *r' *obtained by removing that condition from *r*. Since the premise part of *r' *is less stringent, we obtain that *E(r') *≥ *E(r) *so that the quantity:

(4) $$R\left(c\right)=C\left(r\right)\left(E\left(r^{\prime }\right)-E\left(r\right)\right)$$

can be used as a measure of relevance for the condition *c *of interest.

Since each condition *c *refers to a specific component of ***x***, it is also possible to define a measure of relevance *R_j _*for every input variable *x_j_*:

(5) $$R_{j}=1-\prod_{k}R\left(c_{kl}\right)$$

where the product is computed on the rules *r_k _*that includes a condition *c_kl _*on the variable *x_j_*.

The model *g*(***x***) generated by the LLM task of Rulex can be adopted to produce the output class for any input pattern x^*^, including those that do not verify any generated rule, provided that at least one condition inside at least one rule was verified. To this aim the <*premise*> part of each of the *m *intelligible rules *r_k_*, *k *= 1,..., *m*, describing the model *g*(***x***), is checked to analyze if it is verified by the considered sample x^*^. Let *D*(*r_k_*) be the number of conditions in the <*premise*> part of the rule *r_k _*that are not verified by the pattern x^*^. Then, for every output class *y_l _*we can determine the minimum value $D_{l}={\text{min}}_{r\in G_{l}}D\left(r\right)$ and the subset *H_l _*of rules in the group *G_l _*characterized by that minimum:

(6) $$H_{l}=\left\{r\in G_{l}|D\left(r\right)=D_{l}\right\}$$

Then, we choose as output value for the pattern x^* ^the class *l *scoring the lowest *D_l _*and, in case of ties, the minimum value of the quantity *w_l _*defined as

(7) $$w_{l}=\prod_{r\in H_{l}}\left(1-\sum_{\text{verified}\;c\;\text{in}\;r}R\left(c\right)\right)$$

where the summation is performed on all the conditions *c *in the <*premise*> part of the rule *r *that are verified by the sample x^*^.

### LLM performance assessment

In order to obtain an unbiased estimate of the LLM performance, data were analysed according to a leave-one-out cross-validation (LOOCV). Rules were generated allowing a maximum error rate of 5% in the training set. Accuracy of LLM classification applied to the test set was compared to that of selected competing standard methods of supervised analysis, namely: *k*-Nearest Neighbour classifier (KNN), Artificial Neural Network (ANN), and Decision Tree (DT). In particular, DT, similarly to LLM, is able to generate threshold-based intelligible rules. For this reason, we performed a comparison between the rules generated by LLM and those obtained by DT.

LLM is implemented as part of the Rulex software suite, developed and distributed by RULEX Inc (http://www.rulexinc.com/).

### Competing methods

A brief description of competing methods (KNN, ANN and DT) is here given; details regarding their use and implementation can be found in standard books of data mining [11,12].

#### k-Nearest-Neighbor (KNN)

Although KNN is one of the simplest technique for classifying previously unseen patterns ***x ***taking into account the information contained in a given training set *S*, it can achieve a good accuracy even in complex situations. Its approach is very straightforward: when an input vector ***x ***has to be classified, KNN searches for the *k *nearest points ***x***_1_, ***x***_2_,..., ***x***_k _in *S *according to a given definition of distance. Then, it assigns to ***x ***the most common class present in ***x***_1_, ***x***_2_,..., ***x***_k_. The value of *k *is usually chosen to avoid ties (*e.g*., an odd value for binary classification problems).

Although the adopted definition of distance can affect the accuracy of the KNN classifier, very often the standard Euclidean distance is employed, after having normalized the components of ***x ***to avoid undesirable effects due to unbalanced domain intervals in different input variables. In the reported trials the choice *k *= 1 was performed, which corresponded to assign to any previously unseen point ***x ***the class of its nearest neighbor in the training set *S*.

#### Artificial Neural Network (ANN)

Building a classifier starting from a given training set *S *corresponds to determining a subset of the input domain for each output class or, equivalently, to constructing proper separating surfaces that delimit these subsets. In general, each separating surface can be nonlinear and even complex, depending on the specific classification problem at hand.

A convenient way to manage this complexity is to build the separating surface through the composition of simpler functions. This approach is followed by ANN, a connectionist model formed by the interconnection of simple units, called neurons, arranged in layers. Each neuron performs a weighted sum of its inputs (generated by the previous layer) and applies a proper activation function to obtain the output value that will be propagated to the following layer. The first layer of neurons is fed by the components of the input vector ***x***, whereas the last layer produces the output class to be assigned to ***x***.

Suitable optimization techniques are used to retrieve the weights for each neuron, which form the set of parameters for the ANN. By properly setting these weights we can obtain separating surfaces arbitrarily complex, provided that a sufficient number of neurons is included in the ANN. The choice of this quantity, together with the selection of the number of layers, must be performed at the beginning of the training process and affect the generalisation ability of the resulting model.

#### Decision Trees (DT)

An intelligible classifier can be obtained by generating a tree graph where each node is associated with a condition on a component of the input vector ***x ***(e.g. *x_i _*> 5) and each leaf corresponds to an assignment for the output class to be assigned to ***x***. A model of this kind is called *decision tree*. It is straightforward to retrieve an intelligible rule for the classification problem at hand by navigating the decision tree from a leaf to the root and by using as antecedent for the rule the logical product (AND) of the conditions associated with the nodes encountered during the navigation.

Rules obtained in these way are disjoint from each other.

Although different learning algorithms have been proposed for building a DT, a basic divide-and-conquer strategy is followed by all of them. At each iteration a new node is added to the DT by considering a subset of *S *(generated after previous iterations) and by choosing the condition that subdivides it in the best way, according to a specific measure of goodness. With this approach the size of the subset pertaining to added nodes decreases during the construction of the tree, which halts when a specific stopping criterion is reached (for example all the subsets associated with the leaves are formed by pattern of the same class).

Proper pruning techniques are adopted to simplify the final DT with the aim of reducing its complexity and increasing its generalisation ability.

## Results

### Comparison between the performance of LLM and that of the other supervised methods

Table 2 reports the confusion matrices corresponding to the classification performance on the test set obtained from LLM and from the three competing methods. LLM outperformed any other method of supervised analysis. In fact, accuracy of LLM on the test set was 77.5%, whereas the corresponding figures for the three competing methods were: 72.8% for DT, 54.4% for KNN, and 63.9% for ANN, respectively. In more details, LLM misclassified MPM patients with MTX and with BD approximately at the same rate, while MTX patients were more often misclassified with BD. On the whole, the accuracy evaluated pooling together the two malignancies (the "pooled sensitivity") was 85.1%. DT showed a slightly higher performance than LLM in classifying MPM patients, but a poorer accuracy among MTX and BD classes. Both MPM and MTX patients were more often misclassified with BD. As a consequence, the pooled sensitivity was clearly lower than that estimated during LLM analysis (77.2%). KNN showed a poor accuracy within each considered class. In particular, less than 50% of MTX were correctly identified. However, the pooled sensitivity was 79.8%, slightly higher than that observed for DT, reflecting the fact that most misclassified samples among MPM were allocated to the other class of malignancies (MTX) and vice versa. Finally, for each considered class ANN showed a slightly better performance than KNN but a lower performance than LLM and DT. However, pooled sensitivity was equal to that obtained by DT (77.2%).

**Table 2:** Results of leave-one-out cross-validation. Classification accuracy of 169 patients with pleural disease based on LLM and three considered competing methods.

	Disease status				
		
Classification	MPM N *(%)*	MTX N *(%)*	BD N *(%)*	All N *(%)*	Total Accuracy (%)
*LLM*					77.5
MPM	41 *(78.8)*	9 *(14.5)*	3 *(5.5)*	53 *(31.4)*	
MTX	6 *(11.5)*	41 *(66.1)*	3 *(5.5)*	50 *(29.6)*	
BD	5 *(9.6)*	12 *(19.4)*	49 *(89.1)*	66 *(39.1)*	
*DT*					72.8
MPM	43 *(82.7)*	9 *(14.5)*	5 *(9.1)*	57 *(33.7)*	
MTX	2 *(3.8)*	34 *(54.8)*	4 *(7.3)*	40 *(23.7)*	
BD	7 *(13.5)*	19 *(30.6)*	46 *(83.6)*	72 *(42.6)*	
*KNN*					54.4
MPM	30 *(57.7)*	17 *(27.4)*	7 *(12.7)*	54 *(32.0)*	
MTX	16 *(30.8)*	28 *(45.2)*	14 *(25.5)*	58 *(34.3)*	
BD	6 *(11.5)*	17 *(27.4)*	34 *(61.8)*	57 *(33.7)*	
*ANN*					63.9
MPM	37 *(71.2)*	13 *(21.0)*	12 *(21.8)*	62 *(36.7)*	
MTX	9 *(17.3)*	29 *(46.8)*	1 *(1.8)*	39 *(23.1)*	
BD	6 *(11.5)*	20 *(32.3)*	42 *(76.4)*	68 *(40.2)*	
Total	52	62	55	169	

MPM = Malignant Pleural Mesothelioma; MTX = Metastasis; BD = Benign Diseases; LLM = Logic Learning Machine; DT = Decision Tree; ANN = Artificial Neural Network; KNN = k-Nearest Neighbour Classifier.

### Classification rules obtained by LLM

LLM and DT analyses were repeated on the entire dataset in order to obtain stable rules for patients classification.

LLM classifier included 29 rules, but 15 of them had a very low covering (< 20%).

Table 3 shows the set of the 14 main rules (covering > 20%), while Table 4 reports the corresponding quality measures, according to equations (1) and (4). Four rules were associated to MPM class with a covering ranging from about 54% to 87%. Interestingly, all rules were associated to high values of SMRP and low values of CEA, both identified by different cut-offs. Moreover, high or intermediate values of CYFRA 21-1 were included in three rules. MTX classification was performed by five rules with a covering from 27% to 57%. Three rules included high values of CEA (n. 5, 7 and 8) and among them two were also based on high CYFRA 21-1 concentrations (n. 5 and 8, the latter also including low SMRP values), whereas the remaining rule (n. 7) was associated to a positive CE. The other two rules for MTX classification (n. 6 and 9) both included positive CE and a low or intermediate SMRP value. One of them (n. 6) was also associated to high CYFRA 21-1 concentration. Finally, BD classification was performed by five rules (covering 29% - 71%). Among them, one was based on one condition only (n. 12), corresponding to low values of CYFRA 21-1, while the remaining four were all associated with negative CE and low values of CEA, the latter identified by different thresholds. Two rules also included low values of CYFRA 21-1 (n. 10 and 14), one rule low values of SMRP (n. 13) and the remaining one (n. 11) low values of both CYFRA 21-1 and SMRP.

**Table 3:** LLM classification rules for 169 patients with pleural disease.

n	**Diag**.	1^st ^Condition	2^nd ^Condition	3^rd ^Condition	4^th ^Condition
1	MPM	SMRP > 4.50	CYFRA 21-1 > 71.3	CEA ≤ 8.75	
2	MPM	SMRP > 2.71	88.0 < CYFRA 21-1 ≤ 2518	CEA ≤ 3.75	
3	MPM	SMRP > 1.60	CYFRA 21-1 > 88.0	CEA ≤ 1.55	
4	MPM	SMRP > 17.9	CEA ≤ 2.45		
5	MTX	CYFRA 21-1 > 21.8	CEA > 3.75		
6	MTX	0.58 < SMRP ≤ 25.1	CYFRA 21-1 > 21.8	Positive CE	
7	MTX	CEA > 1.15	Positive CE		
8	MTX	SMRP ≤ 6.88	CYFRA 21-1 > 71.3	CEA > 1.15	
9	MTX	SMRP ≤ 5.26	Positive CE		
10	BD	CYFRA 21-1 ≤ 53.6	CEA ≤ 2.35	Negative CE	
11	BD	SMRP ≤ 3.79	CYFRA 21-1 ≤ 180.6	CEA ≤ 8.00	Negative CE
12	BD	CYFRA 21-1 ≤ 12.7			
13	BD	SMRP ≤ 12.0	CEA ≤ 0.75	Negative CE	
14	BD	CYFRA 21-1 ≤ 86.7	CEA ≤ 0.65	Negative CE	

Diag. = Diagnosis; MPM = Malignant Pleural Mesothelioma; MTX = Metastasis; BD = Benign Diseases (Pleurises); CE = Cytological Examination

**Table 4:** LLM quality measures for the rules shown in Table 3.

		1^st ^Condition		2^nd ^Condition		3^rd ^Condition		4^th ^Condition		
**n**	**Diag**.	**w%**	**R(c)%**	**w%**	**R(c)%**	**w%**	**R(c)%**	**w%**	**R(c)%**	**Cov. %**

1	MPM	14.5	12.6	21.4	18.5	11.1	9.61			86.5
2	MPM	5.13	3.75	30.8	22.5	14.5	10.6			73.1
3	MPM	2.56	1.72	30.8	20.7	23.9	16.1			67.3
4	MPM	59.8	32.2	4.27	2.29					53.8
5	MTX	1.87	1.05	71.0	40.1					56.5
6	MTX	7.48	3.13	0.93	0.38	35.5	14.9			41.9
7	MTX	8.41	3.38	33.6	13.6					40.3
8	MTX	12.2	4.70	11.2	4.33	4.67	1.80			38.7
9	MTX	15.0	4.09	40.2	11.0					27.4
10	BD	29.0	20.5	4.39	3.11	2.63	1.86			70.9
11	BD	18.4	10.7	0.88	0.51	4.39	2.55	3.51	2.04	58.2
12	BD	98.2	37.5							38.2
13	BD	14.9	4.87	23.7	7.74	4.39	1.43			32.7
14	BD	9.65	2.80	16.7	4.85	2.63	0.76			29.1

Diag. = Diagnosis; w% = E(r') - E(r); R(c)% = relevance%; Cov. % = Covering percent. w and R(c) are defined according to equation (4).

### Classification rules obtained by DT

DT classification was based on 8 rules.

**Figure 2:**
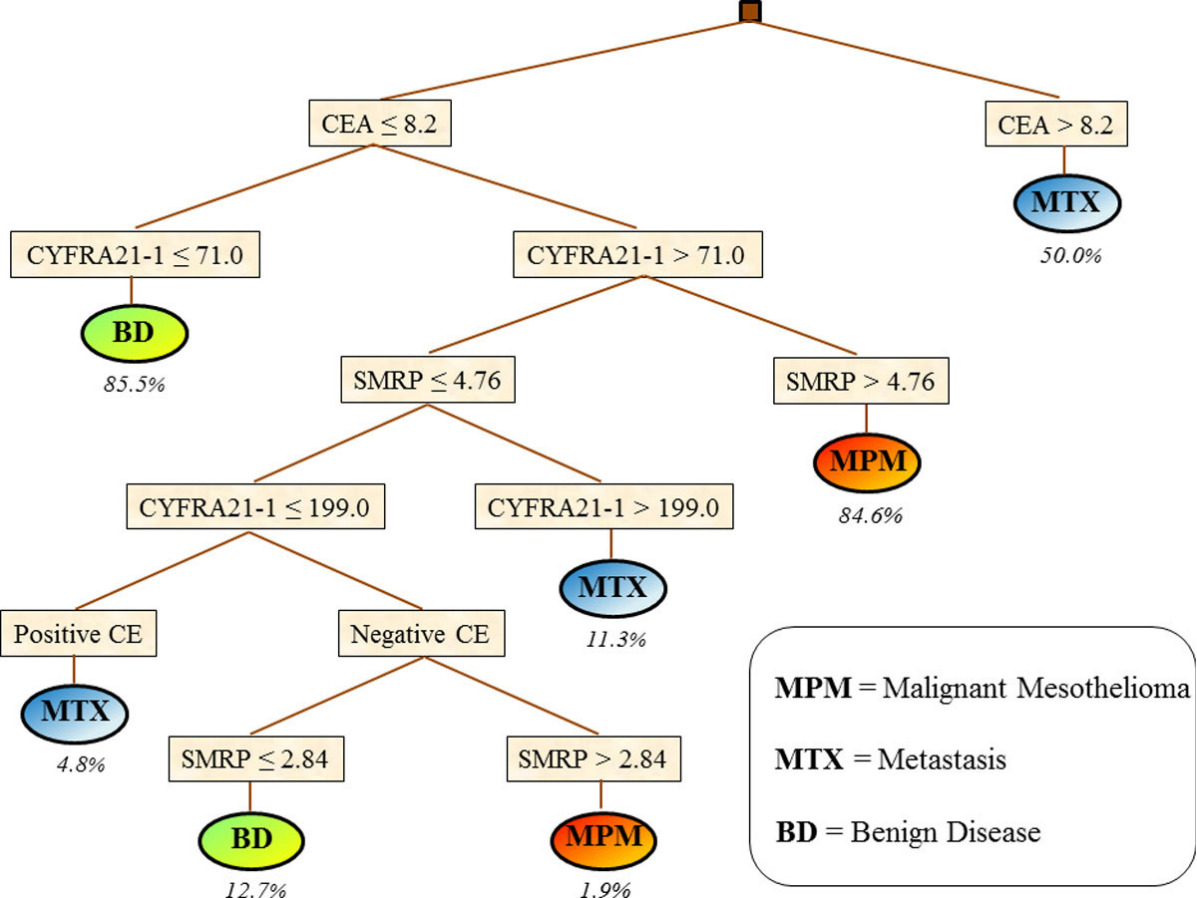
**Classification of 169 patients with pleural disease obtained by Decision Tree**. Percentages indicate the covering of each rule.

Figure 2 shows the DT plot and the corresponding covering of each related rule. MPM classification was based on low values of CEA and high values of both CYFRA 21-1 and SMRP with an 85% covering. MPM was also identified by a more complex rule, based on low values of CEA and SMRP, intermediate values of CYFRA 21-1 and negative CE, but the covering was very low (1.9%), indicating the occurrence of an outlier. MTX patients were identified by three independent rules, namely: a) high values of CEA (covering = 50%); b) low values of CEA associated to high values of CYFRA 21-1 and low values of SMRP (covering = 11%); c) low values of CEA and SMRP, intermediate values of CYFRA 21-1 and positive CE (covering = 5%). Finally, BD classification was based on low values of both CEA and CYFRA 21-1 (covering = 86%), or low values of both CEA and SMRP, intermediate values of CYFRA 21-1 and negative CE (covering = 13%).

## Discussion

LLM is an innovative method that can provide useful classification rules by exploiting the complex multivariable correlation between the different analysed features. LLM has been recently successfully applied to a variety of datasets in biomedical settings [13-17], but so far it has not been used for differential diagnosis of cancer patients based on tumour markers combination.

In the last decades many other methods of supervised data mining have been successfully applied to classification tasks in different biomedical fields, including Oncology. In particular, ANN and KNN have shown a good accuracy in many instances [18]. However, they represent "black-box" methods that cannot provide useful insights about biological and clinical aspects of the disease under study. For this reason, intelligible "AND- type" and "OR-type" rules are in general preferred, but methods for multi-class classification are scarce. Among them, DT is probably the most widely used tool for its simplicity and easily implementation, but in general it tends to show a low accuracy when compared to other supervised methods [18]. However, in the present investigation, DT provided a quite good both total and class-specific accuracy that was higher than that obtained from the two black-box algorithms. However, LLM outperformed all competing methods including DT. In particular, DT performance was slightly lower among BD patients, slightly higher for MPM class only and clearly lower for both MTX and the pool of malignancies.

Classification rules obtained from both LLM and DT were in good agreement with *a priori *knowledge about the considered tumour markers. In particular, high values of CEA were associated with MTX class with a covering of about 50% for both methods (Table 3 and Table 4, rule n. 5 for LLM, and Figure 2, upper side of the plot for DT). Such a proportion roughly corresponds to the percentage of patients with pleural metastasis from lung adenocarcinoma inside the analysed cohort [7]. This finding is in agreement with the characteristics of CEA marker, which is largely expressed among cancers from epithelial origin [4]. Moreover, high SMRP concentrations were associated with MPM classification. This finding confirms previous observations from other independent cohorts reporting that SMRP concentration in pleural fluid is specific in distinguishing mesothelioma from both benign and all other malignant effusions [19,20]. Finally, low values of CYFRA 21-1 were associated with BD classification with a high coverage in both methods (left part of the DT in Figure 2 and rule n. 10 in Table 3), in agreement with previous studies that have associated high values of this marker to a large variety of neoplastic diseases [21].

Rules extracted by LLM and DT only partly overlapped, thus reflecting a sort of balance between the capability of the two methods of identifying useful information for classification purposes, and, on the other hand, the two very different algorithms for rule generation. For instance, the rule with the highest covering for MPM classification was almost identical in the two methods, both including high values of SMRP and CYFRA 21-1 and low values of CEA, with very similar cut-offs. Conversely, the best rule for MTX classification was rather different, including only high values of CEA for DT, and a combination of high values of CEA (at a different cut-off) and high CYFRA 21-1 concentrations for LLM. Finally, the best classification for BD was obtained from both methods by low values of both CYFRA 21-1 and CEA, but at different cut-offs. Furthermore, LLM rule also included negative CE (rule n. 10, Table 3).

On the whole, our results indicate that both LLM and DT are able to extract meaningful information from tumour markers and to combine them in simple rules for classification tasks. DT also provides a simple plot that allows a very easy interpretation of the rules generated, whereas LLM rules, being partly overlapping, provide a rather more complicated picture. However, in our analysis, in agreement with results from previous investigations [13,22] the presence of overlapping rules allowed LLM to outperform DT classification. Furthermore, a non-ambiguous classification can always be obtained by using coverage and error rate parameters and by adopting a proper measure of relevance that allows to select the most probable class for the pattern at hand. Moreover, overlapping rules can be weighted in order to improve classification accuracy in the presence of severely unbalanced sample size [22], thus conferring a high flexibility to LLM based classification.

Results of our investigation should be evaluated at the light of some unavoidable limits, in particular the rather small sample size. Mesothelioma is a rare cancer and, at least at our knowledge, larger datasets including all the three TMs considered in the present study are not available. The possibility that the comparison between the selected classification methods could have been influenced by the size of classes under investigation cannot be completely ruled out. However, in some previous analyses, carried out in different biomedical fields, LLM was demonstrated to outperform other methods of machine learning when applied to large datasets. In particular, LLM accuracy was higher than that of two competing methods (namely: Signal-to-Noise Ratio and Support Vector Machine) in a feature selection task using data from three real and three simulated databases from microarray experiments, each based on many thousands of gene expression profiles [9]. Furthermore, in a recent analysis of biomedical datasets of the Statlog benchmark [23], which included a large database of 268 cases of diabetes and 500 healthy controls, LLM systematically outperformed four competing methods of learning machine (namely: DT, KNN, ANN and binary logistic regression) [17].

Another limit of our investigation is the low accuracy for MTX classification, even if this latter was better classified by LLM than by the considered competing methods. Finally, the set of rules generated by LLM does not cover all the possible combinations between tumour markers and CE results, then making potentially difficult the classification of some additional patients. Such a limitation can be overcome by LLM when the features associated to the subject at least fulfil a subset of one or more conditions inside a composite rule, by combining accuracy measures using equations (6) and (7).

## Conclusions

Results from the present study indicates that LLM is a flexible and powerful tool for the differential classification of malignant mesothelioma patients. DT performance was poorer, but, quite surprisingly, clearly better than the two selected "black-box" competing methods.

Further studies on larger cohorts are needed in order to obtain stable and reproducible rules for MPM classification. Moreover, additional tumour markers should be tested to improve the classification of non-mesothelioma cancers with pleural metastasis.

## List of abbreviations

LLM: Logic Learning Machine.

DT: Decision Tree.

KNN: *k*-Nearest Neighbour classifier.

ANN: Artificial Neural Network.

MPM: Malignant Pleural Mesothelioma.

BD: Benign Disease.

MTX: Metastasis from non-mesothelioma cancers.

CEA: Carcino-Embryonic Antigen.

CYFRA 21-1: soluble fragment of cytokeratin-19

SMRP: Soluble Mesothelin-Related Peptide.

CE: Cytological Examination.

TM: Tumour Marker.

## Competing interests

The authors declare that they have no competing interests.

## Authors' contributions

SP, RF and MM (Muselli) conceived the study and wrote the paper. Moreover, SP performed most analyses, while MM conceived and implemented the LLM method.

PM, RL, GPI and MM (Mussap) provided data of tumour marker concentrations and contributed in the interpretation of biological meaning of results and in writing the Discussion section.

EF, CM and EM implemented most routines for supervised analysis. EF also supervised data analyses.
